# Association of sociodemographic factors with the prescription pattern of opioids for dental patients: A systematic review protocol

**DOI:** 10.1371/journal.pone.0255743

**Published:** 2021-08-05

**Authors:** Alex Junio Silva Cruz, Lucas Guimarães Abreu, Suellen da Rocha Mendes, Lia Silva de Castilho, Mauro Henrique Nogueira Guimarães de Abreu

**Affiliations:** 1 Graduate Programme in Dentistry, School of Dentistry, Universidade Federal de Minas Gerais, Belo Horizonte, Minas Gerais, Brazil; 2 Department of Child’s and Adolescent’s Oral Health, School of Dentistry, Universidade Federal de Minas Gerais, Belo Horizonte, Minas Gerais, Brazil; 3 Department of Operative Dentistry, School of Dentistry, Universidade Federal de Minas Gerais, Belo Horizonte, Minas Gerais, Brazil; 4 Department of Community and Preventive Dentistry, Universidade Federal de Minas Gerais, Belo Horizonte, Minas Gerais, Brazil; University of South Australia, AUSTRALIA

## Abstract

**Introduction:**

Oral health practitioners are responsible for a significant share of opioid prescriptions that seem to be influenced by many aspects, including sociodemographic factors. However, there is no consensus on the factors associated with opioid prescription in Dentistry.

**Objective:**

To identify whether patients’ sociodemographic factors are associated with the prescription pattern of opioids in Dentistry.

**Materials and methods:**

This systematic review will include observational studies (cross-sectional, case-control, and cohort). Electronic searches will be conducted in MEDLINE (PubMed), EMBASE, Scopus, Web of science, LILACS, SciELO, and Google Scholar. Grey literature will also be consulted. Two independent reviewers will screen all retrieved articles for eligibility, extract data, and assess the methodological quality of the included studies. The results will be presented as a narrative synthesis and, where possible, a meta-analysis will be conducted. Certainty of the evidence will be assessed with the Grading of Recommendations, Assessment, Development, and Evaluation approach.

**Systematic review registration number:**

PROSPERO CRD42020211226.

## Introduction

Opioids have long since been used for pain relief, with a key role in modern anesthesia, as well as in postoperative, palliative, and emergency care [1–3]. The analgesia produced by opioids is derived by its complex interaction with receptors of the Central Nervous System [1]. Though effective and largely used worldwide [4–6], opioids are associated with a wide variety of pernicious side effects [1,7]. When administered at higher doses, the probability of addiction and abuse increases over the long-term [8].

The growth in opioid use has led to a health crisis in some countries [9]. In 2017, the United States (US) [10] declared the opioid epidemic a public health emergency. Opioid misuse has been recognized as a national health issue in Australia [11]. The roots of this epidemic rely on the overall recognition that pain has been underestimated by healthcare providers in the past. In 1990, Max [12] stated that pain was being poorly managed and suggested that authorities should encourage the therapeutic use of opioids. In addition, in 1995, pain was described as the fifth vital sign [13]. Associated with pharmaceutical companies’ aggressive marketing of new opioid formulations and other initiatives that came thereafter, healthcare providers became more sensitive in the treatment of pain [9,14]. Consequently, opioid sales skyrocketed [9].

The health and social burdens of an opioid epidemic are significant. From 2005 to 2015, there was a 22.3% increase in disorders associated with opioid use worldwide [15]. In the US, data from 2016 estimated 42,245 deaths provoked by opioid overdose (a mean of 118 deaths daily) [16]. Young adults between 25 and 34 years were the most affected (n = 11,552; 20%), and among individuals within this age range, one in five deaths were associated with opioids [16]. In this scenario, deliberate attempts to mitigate the number of prescriptions of opioids by healthcare providers, including oral health practitioners, have been made [4,9,11].

Oral health practitioners accounted for 8.6% of all providers who prescribe opioids in the US [17]. The prescription patterns of these drugs in Dentistry seems to be influenced by many factors, including the type of procedure performed, the patient’s threshold for pain, and sociodemographic characteristics [18–20]. It seems that women are more likely to receive an opioid prescription [21,22]. However, one systematic review reported that an individual’s sex was not identified as an associated factor for opioid prescriptions after surgery or trauma [23]. Compared to white patients, African-Americans are more likely to receive an opioid prescription provided by a dentist [22,24]. Conversely, another study reported that race was not a predictor for opioid prescriptions in Dentistry [25]. In Brazil, the more privileged the area, the higher the opioid sales [18]. However, in Australia, living in lower socioeconomic conditions was associated with a higher risk of receiving an opioid prescription [6]. Regarding dental insurance, the association with opioid use is still unclear [18,20,22,24].

In view of the opioid epidemic, awareness of the underlying risk factors for opioid prescriptions could contribute to the rational use of this medication [20,23]. Recently, protocols to aid health professionals in opioid prescriptions, such as the Centers for Disease Control and Prevention (CDC) guideline [26], have been provided, but there is still a consensus that a multi-faceted approach is needed to address this issue [27]. Hence, a better knowledge of sociodemographic determinants, associated with the prescription pattern of opioids, may be helpful in the development of interventions to tackle the problem [19,23] and in the reduction of the perpetuated disparities in the use of pain medications between privileged and underprivileged groups [28].

Primary studies have assessed the possible influence of sociodemographic factors on the prescription pattern of opioids in Dentistry; however, the evidence produced by such studies seems controversial [18,20,22,24,25]. A preliminary search was conducted in the PROSPERO, MEDLINE (PubMed), and Web of Science databases, and no systematic review on this issue was identified. Therefore, the objective of this systematic review is to identify if patients’ sociodemographic factors are in fact associated with the prescription pattern of opioids in Dentistry.

## Materials and methods

The proposed systematic review was registered in the International Prospective Register Of Systematic Reviews (PROSPERO): CRD42020211226. The Preferred Reporting Items for Systematic Review and Meta-Analysis Protocols (PRISMA-P) checklist [29] is presented (S1 Checklist).

This study is supported by the Coordination for the Improvement of Higher Education Personnel (CAPES, in Portuguese). This funding agency played no role in the study design, decision to publish, or preparation of the manuscript.

### Review question

Are sociodemographic factors associated with the prescription pattern of opioids for dental patients?

### Inclusion criteria

#### Participants

This systematic review will consider studies with individuals at any age, in which the influence of patients’ sociodemographic factors on receiving an opioid prescription provided by an oral health practitioner was assessed. Studies investigating opioid prescriptions for dental conditions delivered by healthcare providers other than oral health practitioners (e.g., physicians, emergency room [ED] doctors) will be excluded.

#### Exposure

In this systematic review, the exposure will consist of dental patients’ sociodemographic characteristics, including sex, age, race, income, educational level, living environment, and dental insurance. Data sources for the assessment of sociodemographic factors will include validated questionnaires, dental charts/records, and oral healthcare system databases.

#### Outcome

This systematic review will consider the prescription pattern of opioids provided by oral health practitioners as an outcome.

#### Types of studies

Observational studies: cross-sectional, case-control, and cohort studies will be included. Letters to the editor, editorials, ecological studies, case reports, case series, and literature reviews will be excluded.

### Search strategy

For the definition of the search strategy for this systematic review, three steps were taken. First, a preliminary search was performed in MEDLINE (PubMed) to identify articles that meet the inclusion criteria. Thereafter, the titles and abstracts of these studies were used to identify keywords and indexing terms to develop the final search strategy. Second, with the assistance of a librarian, the final search strategy was tailored for MEDLINE (PubMed) using MeSH terms, entry terms, and synonyms linked with Boolean operators (S1 File). The search was adapted for each of the other databases, using controlled vocabulary (MeSH, EMTREE, and others), as well as entry terms.

Finally, the list of references of all studies included in the systematic review will be screened. Before final analysis, the searches will be updated. No restrictions will be applied to language and year of publication, or geographic limits.

#### Information sources

Searches will be conducted in the following electronic databases: MEDLINE (PubMed), EMBASE, Scopus, Web of Science, LILACS and SciELO. A search in Google Scholar, limited to the first 200 most relevant studies [30], will also be conducted. Grey literature will be searched in OpenGrey.

### Study selection

All identified citations will be uploaded into EndNote 20 (Clarivate Analytics, PA, USA) and duplicates removed. Two reviewers will select the studies independently. They will begin by assessing the titles/abstracts. Those that meet the inclusion criteria will be selected. If the title/abstract does not provide sufficient information for a decision, the full text of the reference will be evaluated. References whose full text fulfills the eligibility criteria will be included. Any disagreements arising during the study selection will be discussed and resolved by consensus. If an agreement is not reached, a third reviewer will decide. Interrater reliability will be estimated with the kappa test.

### Data extraction

Two reviewers will extract all data from the included studies independently and in duplicate. If divergences between reviewers occur, a discussion will be set in place until a consensus is reached. Data extracted will include study details (author, year, journal), study methods (design, setting, sample, recruitment process, exposure), prescription of opioids (frequency, dose, duration of prescribed opioid), and the results of evaluation of the association of exposures (sociodemographic factors: sex, age, race, income, educational level, living environment, and dental insurance) with opioid prescriptions. The form developed for data extraction is displayed as supporting information (S2 File).

### Assessment of methodological quality

Two reviewers will assess the methodological quality of the included studies independently. Disagreements arising during this process will be discussed and resolved by consensus. In the cases in which agreement is unattainable, a third reviewer will be consulted. Depending on each study design, the following JBI’s critical appraisal tools will be deployed: Checklist for Analytical Cross-Sectional Studies, Checklist for Case Control Studies, and Checklist for Cohort Studies will be used, depending on each study design [31].

In the cross-sectional studies, eight items will be assessed: definition of the inclusion criteria; depicted information of participants, study´s setting, and time period; use of valid and reliable methods for the assessment of the exposure; if objective and standard criteria were employed to assess the condition; awareness of confounders; reliable measurement of the outcome; and use of adequate statistical analysis [31].

In the case control studies, 11 items will be assessed: similarity between groups, presence of disease in cases or the absence of disease in controls; matching of cases and controls; adoption of the same criteria to identify cases and controls; adoption of a valid and reliable strategy for the measurement of the exposure; same measurement of the exposure for cases and controls; awareness of confounders; strategies to handle confounders; adoption of a valid and reliable method for the measurement of the outcome for cases and controls; period of interest of the exposure, if this period was long enough to be significant; and adoption of adequate statistical analysis [31].

In the cohort studies, 11 items will also be assessed: recruitment of participants of the two groups from the same population; similar measurement of the exposure to assign participants to both exposed and unexposed groups; use of a valid and reliable instrument for the measurement of the exposure; identification of confounders; strategies to deal with confounders; groups of individuals without the outcome at the study’s onset; adoption of a valid and reliable strategy for the measurement of the outcome; report of the follow-up period and whether this period is long enough for the occurrence of the outcome; information whether follow-up was complete or at least a statement on the reasons for follow-up losses; strategies to handle incomplete follow-up; and adoption of adequate statistical analysis [31].

In each study, three ratings may be assigned to the items: ‘low risk of bias’ (if the answer to the question is ‘Yes’), ‘high risk of bias’ (if the answer to the question is ‘No’), and ‘unclear risk of bias’ [31].

All studies will be submitted to data extraction and synthesis. The impact of risk of bias will be considered when developing conclusions and recommendations (if feasible).

### Data synthesis

In the first step of data synthesis, we will present the results of the study selection process using the PRISMA [32] statement flowchart. The interrater reliability (agreement between the two reviewers) estimate will be given with the kappa test.

In the second step of data synthesis, homogeneous data will be aggregated into meta-analyses. Effect measures will be expressed as either odds ratio/risk ratio (for dichotomous data), or mean differences (for continuous data). In the case of meta-analyses of continuous outcomes, for which included studies used different scales, the standardized mean difference will be determined. The 95% confidence interval (CI) will be calculated as well. The possibility of combining studies of continuous and dichotomous outcomes through transformations will be assessed. Heterogeneity will be assessed using chi-square and the I^2^ statistic [33]. The confidence interval of the I^2^ will be determined as well. The following formula will be used: *exp*(ln *I*^2^±1.96×*SE*[*ln*(*I*^2^)]) [34]. The random effect model will be deployed in the meta-analysis [35]. We will also check whether the results of the meta-analyses remain even if undetected heterogeneity is assumed. The random effect model will be implemented through the DerSimonian and Laird inverse variance [33]. Meta-analyses will be developed at RevMan 5.4 (Copenhagen, The Nordic Cochrane Centre, Cochrane).

Sensitivity analysis might be conducted with the removal of studies one by one in an attempt to reduce heterogeneity. If possible, subgroup analyses will be performed according to the different sociodemographic factors assessed (sex, age, race, income, educational level, living environment, and dental insurance). The design of the included studies will also be considered during the analysis of subgroups. The results of meta-analysis will be presented as forest plots.

Publication bias will be addressed by developing a funnel plot [33]. The RevMan 5.4 (Copenhagen, The Nordic Cochrane Centre, Cochrane) will be used. Considering that the visual evaluation of the funnel plot might be subjective, we will run a test for funnel plot asymmetry, so long as at least ten studies are incorporated into the meta-analysis [33,36]. The regression method for the detection of funnel plot asymmetry proposed by Harbord and colleagues (2006) [37] will be used for dichotomous outcomes. For the meta-analysis of continuous outcomes, the assessment of funnel plot asymmetry will be performed with the Egger test [38]. Moreover, if less than ten studies are incorporated into the meta-analysis, no funnel plot will be constructed, and this will be reported and discussed as a limitation of the systematic review. Bias assessment, considering the year of publication of the articles, will also be performed, as publication bias in systematic reviews of more recent studies is lower [39].

### Certainty of evidence

If there is sufficient evidence to make recommendations, the Grading of Recommendations, Assessment, Development and Evaluation (GRADE) [40] will be used to rank the certainty of evidence. The GRADEpro (McMaster University, ON, Canada) will be used to construct a Summary of Findings. GRADE is based on five domains: the risk of bias within individual studies, the study design, the indirectness of the evidence, the inconsistency, the imprecision of the effect size estimates, and the risk of publication bias. The certainty of the evidence for each outcome may be ‘very low’, ‘low’, ‘moderate’, or ‘high’ [40].

## Supporting information

S1 Checklist PRISMA-P 2015 checklist: Recommended items to address in a systematic review protocol. (PDF)

S1 File Search conducted in MEDLINE (PubMed) on September 28th, 2020. (PDF)

S2 File Form to extract data of included studies. (PDF)
