# Supplementary material for: Association of sociodemographic factors with the prescription pattern of opioids for dental patients: A systematic review protocol
Source: PLoS One. 2021 Aug 5;16(8):e0255743. doi: 10.1371/journal.pone.0255743 (PMC8341526; doi:10.1371/journal.pone.0255743)
Supplement: S1 File — (PDF) [file pone.0255743.s002.pdf]

S1 File. Search conducted in MEDLINE (PubMed) on September 28th, 2020.

Search	Query	Record retrieved
#1	Dentistry [MeSH Terms] OR "Dental Care"[MeSH Terms] OR “Dental Treatment” OR “Dental Procedures” OR "Dental Patient" OR "Dental Patients" OR "Dental Outpatient" OR "Dental Outpatients" OR “Oral Health Care” OR “Oral Health Service” OR “Dental Health Services” [MeSH Terms]	418,054
#2	“Socioeconomic Factors”[MeSH Terms] OR “Sociodemographic Characteristic” OR "Demography Factors" OR "Healthcare Disparities”[MeSH Terms] OR “Social Class”[MeSH Terms] OR "Social Conditions"[MeSH Terms] OR Sex[MeSH Terms] OR Gender OR "Age Groups"[MeSH Terms] OR "Age Group" OR Age OR "Race Factors"[MeSH Terms] OR Race OR Ethnicity OR “Skin Color” OR “Skin Colour” OR "Educational Status"[MeSH Terms] OR "Education Level" OR Schooling OR Income[MeSH Terms] OR Wage OR Insurance[MeSH Terms] OR "Insurance, Dental"[MeSH Terms] OR "Dental Insurance" OR "Plan, Dental Care" OR "Living area" OR “Residence Area” OR Residence	14,143,410
#3	"Analgesics, Opioid"[MeSH Terms] OR "Opioid Analgesics" OR Opioid OR Opioids OR Narcotics[MeSH Terms] OR “Narcotic Analgesics” OR “Analgesics, Narcotic” OR “Opioid Prescription” OR “Opioids Prescriptions” OR “Opioids Prescribing” OR “Prescribing Opioids” OR “Prescribed Opioids” OR “Painkiller” OR “Painkillers” OR “Pain-killers” OR Morphine[MeSH Terms] OR “Morphine Derivatives”[MeSH Terms] OR Codeine[MeSH Terms] OR Oxycodone[MeSH Terms] OR Tramadol OR Hydrocodone[MeSH Terms] OR Fentanyl[MeSH Terms]	184,913
#4	((#1) AND (#2) AND (#3))	933
