# Supplementary material for: Association of sociodemographic factors with the prescription pattern of opioids for dental patients: A systematic review protocol
Source: PLoS One. 2021 Aug 5;16(8):e0255743. doi: 10.1371/journal.pone.0255743 (PMC8341526; doi:10.1371/journal.pone.0255743)
Supplement: S2 File — (PDF) [file pone.0255743.s003.pdf]

S2 File. Form to extract data of included studies.

Reviewer: ____________________ Date: ________________

Data extraction form

**Study details**			
First author:			
Year:			
Journal:			
**Study method/ characteristics**			
Study design:	( ) Cross sectional	( ) Case control	( ) Cohort
Setting:			
**Participants**			
Sample size:			
Age (mean):			
Sex:	Male (n;%)	Female (n;%)	
Country/ location:			
Recruitment procedures:			
Follow-up or study duration:			
Exposure(s) of interest:			
**Dependent variable (outcome)**			
Opioid prescriptions:	Yes (n;%)	No (n;%)	
Frequency of prescribed opioids:			
Dose and duration of prescribed opioids:			
Author's conclusion:			

Data analysis

Covariates	Effect measure	IC	p-value
**Sex**			
Male			
Female			
**Age**			

**Race**
**Income**
**Educational level**
**Living environment**
**Dental insurance**
No
Yes

Statistical analysis: ____

Effect measure:	( ) Risk ratio	( ) Odds ratio	( ) Other:_________
IC:	( ) 95%	( ) Other: _________	

Reviewer comments

____

____

____
